# A case report of sinusoidal diffuse large B-cell lymphoma in a STK4 deficient patient

**DOI:** 10.1097/MD.0000000000018601

**Published:** 2020-02-28

**Authors:** Farzaneh Ashrafi, Christoph Klein, Mohaddese Poorpooneh, Roya Sherkat, Razieh Khoshnevisan

**Affiliations:** a Internal Medicine Department, Isfahan University of Medical Sciences, Isfahan, Iran; b Department of Pediatrics, Dr. von Hauner Children's Hospital, University Hospital, LMU Munich, Munich, Germany; c Acquired Immunodeficiency Research Center, Isfahan University of Medical Sciences; d Immunology Department, Isfahan University of Medical Sciences, Isfahan, Iran.

**Keywords:** immunodeficiency, lymphoma, STK4

## Abstract

**Introduction:**

Primary immunodeficiency diseases (PIDs), a rare group of gene defects with different manifestations, are at great risk of malignancy. The incidence of diffuse large B-cell lymphoma in the sinusoidal tract is quite rare with nasal congestion, stuffiness, and pain in maxillary sinus manifestation. Human serine-threonine kinase 4 (STK4) deficiency affects the immune system with recurrent bacterial and viral infections, mucocutaneous candidiasis, cutaneous warts, skin abscesses, T- and B-cell lymphopenia, and neutropenia.

**Patient concern:**

In this study we describe the infrequent incidence and successful treatment of sinusoidal diffuse large B-cell lymphoma in a STK4 deficient patient with clinical manifestation of severe intractable headaches, unilateral swelling of her face, nasal congestion, stuffiness, and pain in maxillary.

**Diagnosis:**

Clinical data including headaches, unilateral swelling of face, nasal congestion, stuffiness and pain in maxillary sinus with confirmed histopathology and magnetic resonance imaging finding confirmed sinusoidal diffuse large B cell lymphoma in a STK4 deficient patient.

**Intervention:**

Six cycles of R-CHOP (rituximab with cyclophosphamide, doxorubicin, vincristine, and prednisolone) were administered and after each cycle, G-CSF support was used. Chemotherapeutic drugs were administered with standard dose and no dose reduction was done during the treatment. IVIG treatment continued during the courses of chemotherapy.

**Outcome:**

The index patient achieved complete response at the end of chemotherapy courses and was in remission for about 8 months afterward, prior to the date of the present report.

**Conclusion:**

PID patient are often at increased risk of malignancies. Sinusoidal diffuse large B-cell lymphoma is quite rare and prognosis is variable. Early attention to patient's manifestation, suitable treatment, and monitoring manifestations caused by PID are critical.

## 1 Introduction

Primary immunodeficiency diseases (PIDs) is a rare group of >350 single gene defects affecting the immune system^[1]^ with an enhanced susceptibility to specific infections and an increased incidence of malignancy.^[2]^ Diffuse large B-cell lymphoma (DLBCL) is the most common B-cell malignancy in patients with PID.^[3,4]^ The paranasal sinus is very rarely affected in extra nodal lymphoma.^[5]^

Serine-threonine kinase 4 (STK4) is the mammalian homolog of Drosophila Hppo protein, controlling cell growth, apoptosis, tumorigenesis,^[6]^ which also has a critical role in unrestricted EBV-induced lymphoproliferation.^[7]^

STK4 loss in the mouse chemically and genetically enhanced lymphoma development by inducing chromosomal instability.^[8]^ Human STK4 deficiency as a PID syndrome functionally affects T cells, B cells, and neutrophil.^[9]^ Advances in PID diagnosis and supportive management have led to an increase in life expectancy. Treatment strategy in PID is a challenging clinical issue in the condition of limited therapy tolerance, infectious condition, and tumor status.^[10]^

In this report, we describe the first case of maxillary sinus DLBCL, which has been diagnosed as a novel PID syndrome, STK4 deficiency.

## 2 Case presentation

**Figure 1:**
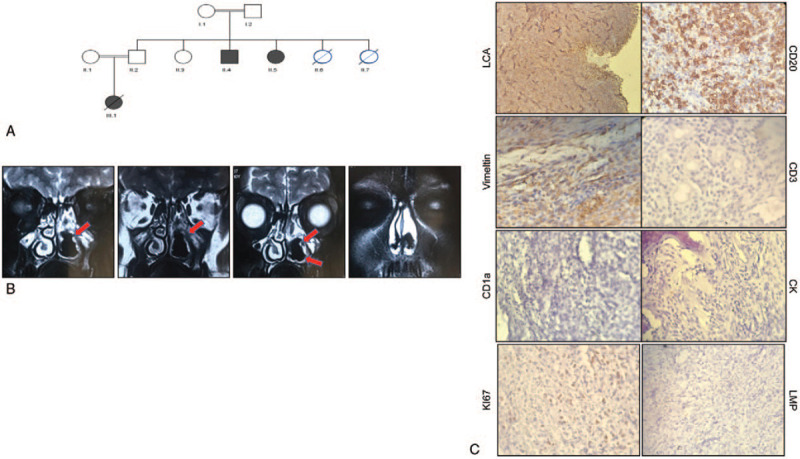
(A) Pedigree of the index family with three confirmed STK4 deficiency patients (II-4, II-5, III-1). II-5 is the patient who was described in this paper, III-3 was deceased because of primary cardiac T-cell lymphoma. (B) MRI study showed left maxillary sinus as well as nasal cavity are completely opacified with mass lesion. (C) Immunohistochemistry result in sinus biopsy indicated positive in LCA, CD20, Vimeltin and negative for CD3, CD11a, CK, KI67, and LMP. STK4 = serine-threonine kinase 4.

A 32-year-old female patient from a consanguineous Iranian (Fig. 1A) family was originally presented with recurrent mouth ulcers, upper and lower respiratory tract infections (otitis, fungal and bacterial sinusitis, lung infection), vaginal ulcers and abscesses, mucocutaneous candidiasis, skin abscesses, fever, disseminated warts (induced by molloscum cantagiosum), and severe pneumonia. The patient belonged to a family in which human STK4 deficiency had originally been reported (previously published).^[9]^ She had severe periodic neutropenia (600–1700/μL), episodic leukopenia (white blood cells 750–4430/μL, lymphocytes: 266–821/μL, CD3: 177/μL, CD4: 120/μL, CD8: 86/μL, CD19: 57/μL, CD56: 49/μL), high-IgG level (27.1 g/L), and low-IgM level (0.22 g/L) since childhood, receiving treatment with intravenous immunoglobulin (IVIg) 500 mg/kg, on a 3-week regular basis accompanied by clinical follow-up exams.^[11]^

In three continuous visits with the patient, she complained of severe intractable headaches, unilateral swelling of her face, nasal congestion, stuffiness, and pain in maxillary sinus, all of which abided despite symptomatic therapy. In view of the mentioned persistent symptoms, imaging studies were carried out.

Magnetic resonance imaging (MRI) showed a mass-like opacification in the right nasal cavity extending along maxillary sinus with reactive sclerosis in sinus wall (Fig. 1B). This feature was mentioned to be due to chronic fungal infection or malignancy; consequently, sinus endoscopy & biopsy were done. Pathology showed neoplastic proliferation of monotonous lymphoid cells in diffuse pattern with high N/C ratio, large vesicular nuclei and high mitotic figures, which was in favor of non-Hodgkin lymphoma (NHL). Immunohistochemistry (IHC) staining proved positive for LCA, CD20, Vimentin and negative for CD3, CD10, CD5, and CK, confirming high-grade DLBCL (Fig. 1C).

In staging, no other site of involvement was detected and cerebrospinal fluid (CSF) analysis was negative for malignancy. Moreover, primary biochemical tests were ran and, normal renal and hepatic function being ensured, chemotherapy was started. Chemotherapy regimen include six cycles of R-CHOP (rituximab with cyclophosphamide, doxorubicin, vincristine, and prednisolone) with 3-week intervals were administered and after each cycle, G-CSF support was used. Chemotherapeutic drugs were administered with standard dose and no dose reduction was done during the treatment. IVIG treatment continued during the courses of chemotherapy. Accordingly, the patient had no episode of fever and neutropenia or other complications of chemotherapy. The patient achieved complete response at the end of chemotherapy courses and was in remission for about 8 months afterward, prior to the date of the present report.

## 3 Discussion

In this report, we describe a rare case of maxillary sinus DLBCL in a 32-year-old STK4-deficient woman.

DLBCL is the most common subtype of NHL and comprises a heterogeneous group of tumors that differ at clinical, pathological, molecular, and biological levels^[12]^ – an aggressive NHL that may arise inside or outside the lymphatic system. Gastrointestinal (GI) tract, skin, bone, and brain are the most common sites of extra nodal lymphoma locations.^[13]^ The incidence of NHL in males is significantly higher than in females.^[14]^ NHLs detected in the sinusoidal tract are quite rare, and represent only 3% to 5% of all malignancies.^[15]^ Squamous cell carcinoma had been reported as the predominant epithelial cell type (80%) followed by adenocarcinoma type (10–20%) of all sinusoidal malignancies.^[16]^

Around 55% of sinusoidal tumors originate from the maxillary sinus, 35% from the nasal cavity, 9% from the ethmoid sinus, and the remainder from the frontal and sphenoid sinuses.^[16]^

Clinical presentations of sinus lymphoma are variable and range from shortness of breath, wheezing, high-pitched breathing sounds in low grade lymphoma to non-healing ulcer, cranial nerve manifestations, facial swelling, epistaxis, or pain in high-grade lymphoma.^[17]^ Early stages of sinusoidal tumor are often asymptomatic or mimicking inflammatory diseases.^[18]^

Radiographic examination, including CT or MRI must be conducted to determine the location, shape, and size of NHL in sinus and also differentiate NHL from squamous cell carcinoma by an evaluation of bone destruction.^[19]^ Immunohistochemical studies are warranted to clear histopathological diagnosis. NHL usually express pan B-cell markers such as CD19, CD20, CD75, CD79a, and CD22, but may lack one or more of them.^[20]^

Advances in diagnostic procedure, supportive treatment and novel therapeutic agents have resulted in a significant improvement in the survival of patients with DLBCL.^[21]^ Although most patients respond to six to eightcycles of R-CHOP chemotherapy treatment regimen, about 10% to 15% has primary refractory disease and a further 20% to 30% relapse.^[22]^

Human STK4 deficiency is a primary immunodeficiency syndrome associated with recurrent bacterial and viral infections, mucocutaneous candidiasis, cutaneous warts, and skin abscesses. T- and B-cell lymphopenia and neutropenia are reported as immunophenotyping phenotype of STK4 deficient patients.^[9]^

STK4 is a key kinase in the HIPPO pathway, which regulates tissue growth control, apoptosis, and tumorigenesis in mammalians,^[23]^ and its activity can be regulated by caspase-induced cleavage, prompting translocation to the nucleus, and mainly interaction with Yes-associated protein (Yap) and then activation of TEAD1-4 transcription factors.^[8,24]^ Five out of 16 patients with reported STK4 deficiency were presented with EBV-driven lymphoproliferation.^[25]^ STK4 has a critical role in the control of unrestricted EBV-induced lymphoproliferation and is necessary for FOXO1 and FOXO3 activation which is a crucial transcription factor for cytotoxic T-cell response to chronic viral infection.^[7]^

Stk4−/− mice lymphocytes are prone to mis-segregate chromosomes during cell cycle and indicate enhanced levels of aneuploidy, which is likely to cause tumorigenesis.^[8]^ Furthermore, a decrease in the count of T and B cells was reported in stk4 knock-out mice base on defect in pro-apoptotic role of stk4.^[9]^ In addition, STK4 expression was significantly reduced in different B-cell lymphoma such as DLBCL, follicular lymphoma, Burkitt lymphoma, hairy cell leukemia, mantle cell lymphoma, and primary effusion lymphoma. This suggests that STK4 insufficiency is critical in the pathogenesis of precursor lymphoblastic leukemia and mature lymphocyte neoplasms in humans.^[8]^ Stk4-deficient mice exhibited reduced BCR signaling, which is related with defective BCR clustering and causes a defect in the differentiation of marginal zone and germinal center.^[8]^ Interestingly, loss of STK4 in human leads to the imbalance in T-cell signaling and increased apoptosis and decreased count of T cells.^[26]^

Our STK4-deficient patient with a rare sinusodial DLBCL highlights the need to consider unusual malignancies. Even though the incidence of sinusodial DLBCL is quite rare, clinicians should be aware and differentiate between inflammatory manifestation and malignancies in sinus.

## 4 Conclusion

PID patient are often at increased risk of malignancies, mostly NHL. Sinusoidal DLBCL is quite rare and prognosis is variable, dependent on the stage and aggressiveness of the tumor. Early attention to patient's manifestation, doing biopsy surgery, pathological examination, suitable chemotherapy regimen, and monitoring manifestations caused by PID are required for diagnosis and treatment.

## Acknowledgments

We would like to thank the patients and their families for their collaboration and their participation in the present study.

## Author contributions

**Conceptualization:** Roya Sherkat.

**Data curation:** Razieh Khoshnevisan.

**Investigation:** Christoph Klein, Razieh Khoshnevisan.

**Methodology:** Farzaneh Ashrafi, Roya Sherkat.

**Project administration:** Farzaneh Ashrafi, Christoph Klein.

**Supervision:** Razieh Khoshnevisan.

**Validation:** Mohaddese Poorpooneh, Razieh Khoshnevisan.

**Visualization:** Mohaddese Poorpooneh.

**Writing – original draft:** Roya Sherkat, Razieh Khoshnevisan.

**Writing – review & editing:** Christoph Klein.
